# Root Extracts of Two Cultivars of *Paeonia* Species: Lipid Composition and Biological Effects on Different Cell Lines: Preliminary Results

**DOI:** 10.3390/molecules26030655

**Published:** 2021-01-27

**Authors:** Natalia Calonghi, Giovanna Farruggia, Carla Boga, Gabriele Micheletti, Elena Fini, Lucia Romani, Dario Telese, Erika Faraci, Christian Bergamini, Stefano Cerini, Nicola Rizzardi

**Affiliations:** 1 Department of Pharmacy and Biotechnology, University of Bologna, Via Irnerio 48, 40126 Bologna, Italy; giovanna.farruggia@unibo.it (G.F.); erika.faraci@hotmail.it (E.F.); christian.bergamini2@unibo.it (C.B.); nicola.rizzardi2@unibo.it (N.R.); 2 National Institute of Biostructures and Biosystems, Via delle Medaglie D’oro 305, 00136 Roma, Italy; 3 Department of Industrial Chemistry ‘Toso Montanari’, Alma Mater Studiorum Università di Bologna Viale Del Risorgimento 4, 402136 Bologna, Italy; gabriele.micheletti3@unibo.it (G.M.); elena.fini2@studio.unibo.it (E.F.); dario.telese2@unibo.it (D.T.); stefano.cerini3@unibo.it (S.C.); 4 Via Riniera 2043, 40024 Castel San Pietro Terme, Italy; luciaromani@virgilio.it

**Keywords:** *Paeonia*, root extraction, QPI, ROS, cancer, mitochondria

## Abstract

The roots of two cultivars of *Paeonia*, namely *Paeonia officinalis* “Rubra Plena” and *Paeonia* “Pink Hawaiian Coral”, have been extracted with chloroform. The composition of the lipid fraction, analyzed by GC–MS technique, revealed the absence of paeonol and the presence of phenol, benzoic acid, fatty acid—and some sterol—derivatives. The chloroformic extracts have been tested on normal and several cancer cell lines but showed antiproliferative activity only on the ovarian carcinoma and the osteosarcoma. The biological activity of extracts was investigated mainly by confocal microscopy, flow cytometry and quantum phase imaging. The results indicated that the root extracts induced a hyperpolarization of mitochondria and an increase in reactive oxygen species levels, without inducing cell death. These effects are associated to an increased doubling time and a retarded confluence.

## 1. Introduction

The genus *Paeonia* L. (Paeoniaceae family) comprises about 35 species divided into two groups: the herbaceous kinds and the tree peony [1,2].

Herbaceous peonies are perennial herbs that comprise 24 wild species distributed in the temperate climates of Eurasia, northwestern Africa, and North America, and eight species with four subspecies widely distributed in China [3,4].

Many species of this single genus have been used for thousands of years in traditional medicine. It was mentioned in ancient Greek and Roman herbal remedies, and used in the Middle Ages for curing epilepsy, bladder stones, jaundice, stomachache, diarrhea, labor pains, nightmares, and lunacy [5,6,7]. However, until now, studies on the biological activity of *Paeonia* have been carried out mainly in species typical of the Asian continent, especially of China [8,9,10], whereas scientific reports for *Paeonia officinalis* L. are more scarce [11,12,13].

Two kinds of herbaceous peony roots, namely, white and red peony roots, are used for different remedies in traditional Chinese medicine [14]. The difference between the two types of roots has been debated for a long time. An interesting study analyzed the nucleotide sequence of DNA and the contents of eight main bioactive constituents obtained from different *Paeonia* species, demonstrating that white and red *Paeonia* roots were not only geographically isolated, but also genetically and chemically separated [15].

Actually, phytochemical investigations have demonstrated that the bioactive compounds contained in paeony roots are predominantly monoterpenoids, tannins and paeonols, which have been shown to possess a variety of biological activities, such as anti-inflammatory, immunomodulatory, antitumor, antiviral, antibacterial, antifungal, antioxidant and pro-oxidant, hepatoprotective, and neuroprotective effects [1,16,17,18].

Monoterpene glycosides, such as paeoniflorin, albiflorin, oxypaeoniflorin, benzoylpaeoniflorin, and benzoylalbiflorin, are the major characteristic compounds of *Paeonia* roots. Paeoniflorin is the most abundant compound reported in both white and red *Paeonia* roots, which is also unique to the genus *Paeonia*. Research has also demonstrated various activities of paeoniflorin and its isomer albiflorin, such as anti-inflammatory, hepatoprotective [18], and neuroprotective effects [19]. Total glucosides of *Paeony*, a mixture mainly composed of paeoniflorin and albiflorin, was approved by the China Food and Drug Administration for the treatment of rheumatoid arthritis [20].

Gallic acid and pentagalloylglucose are abundant hydrolyzable tannins found in both white and red *Paeonia* roots [18]. Gallic acid, a polyphenol not unique to *Paeonia* species, has been extensively studied for its antioxidant and antiviral activity [18]. Moreover, various activities of pentagalloylglucose have been reported, including anti-inflammatory, anti-allergic, antitumor, antiviral, and antibacterial effects [18].

Paeonol is a compound commonly found in the genus *Paeonia*; it has been highly investigated for anti-inflammatory and antitumor activities, while other pharmacological activities such as anti-allergic, antioxidant, glycemic, cardiovascular and neuroprotective effects have also been reported [17].

Benzoic acid is well known for its antifungal activity and it is found in many plants including both *P. lactiflora* and *P. veitchii* [21,22].

Oxidative activity has been largely focused on *P. lactiflora* constituents and the mechanisms for reduced damage from oxidative species and/or enhanced scavenging of these species has been investigated. The monoterpene glycoside albiflorin, gallic acid, paeonol, paeonilactone C, palbinone, show antioxidant activity with different mechanisms [18].

Other compounds, such as tellimagrandin I, show antioxidant activity at low concentration, while at high concentration they can have pro-oxidant activity [23,24].

However, apart a few reports [25,26], until now almost all scientific studies on the biological activity of *Paeonia* species roots have been carried out on alcoholic [27,28,29,30] or aqueous extracts [11].

Based on the above, and considering that in many countries of Europe and regions of Italy [31] wild paeonies are protected species, with the aim to gain novel information pursuing the principles of an environmentally friendly approach, we decided to start an investigation on the biological activity of the lipidic fraction obtained by extraction with chloroform of roots of two commercially available cultivars of *Paeonia*, namely *Paeonia officinalis* “Rubra Plena” and *Paeonia* “Pink Hawaiian Coral”. Moreover, we chose these two cultivars for their propensity to produce adventitious buds from even small portions of rhizome, a feature that might be useful to obtain considerable amounts of roots easily.

## 2. Results and Discussion

### 2.1. Chemistry

#### Extraction Results

**Figure 1:**
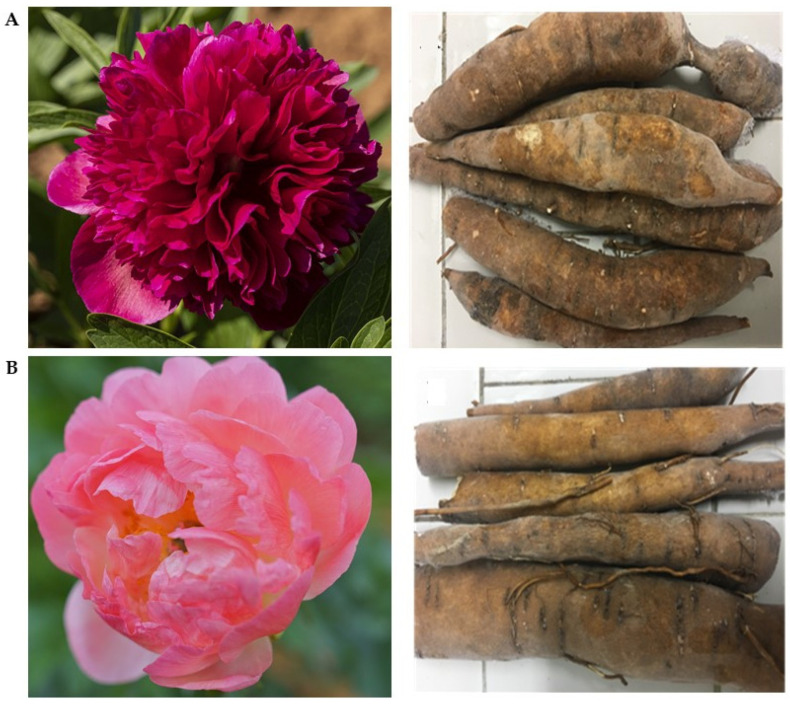
(**A**) Flower and roots of *Paeonia officinalis* “Rubra Plena” and (**B**) Flower and roots of *Paeonia* “Pink Hawaiian Coral”.

The roots of the two cultivars (Figure 1) were washed with water, finely cut and dehydrated in oven at 70 °C, and then reduced in powder, obtaining 42% and 39% of weight yield from “Rubra Plena” and “Pink Hawaiian Coral”, respectively.

The same amount of the powdered dried roots of the two cultivars were then subjected to continuous solid–liquid extraction with chloroform. After 24 h, the solvent was removed and in both cases the resulting residue was recovered in 0.6% yield with respect to the weight of starting dried powdered roots. The chloroformic extracts derived from “Rubra Plena” and “Pink Hawaiian Coral” roots are hereinafter indicated as **SCO** and **SCH**, respectively.

Equal amounts of the two extracts were subjected to the same dilution (see experimental) and analyzed by GC–MS. The main components were identified by comparison to retention times and mass spectra of known compounds or by comparison of the spectra based on the similarity with Wiley275L mass library data (only spectra showing with a similarity greater than 87% were taken into consideration). We considered the peak area by GC–MS of the two extracts (obtained and treated rigorously in the same manner) in order to compare their content. In Table 1, the names of common components present in the chloroformic extracts of the roots of the two *Paeonia* varieties and the relative percentage based on the area of the chromatographic peak are reported.

As can be seen, the **SCH** has a higher benzoic acid, phenol, palmitic acid, and oleic acid content with respect to **SCO**. Opposite behavior is presented for the content of linoleic acid, methyl linolenate, and vitamin E. It is noteworthy that, while benzoic acid is known to be widely distributed in *Paeonia* plants including roots [17], the above cited fatty acids, including margaric acid (i.e., heptadecanoic acid, found in *Paeonia decora*), have been found mainly in fresh seeds [17].

Among compounds characterized by steroidal scaffold, a derivative with mass spectrum very similar to that of aplysteryl compounds has been detected in both samples, **SCO** and **SCH**. It has to be noted that the presence of aplysteryl derivatives has been previously found in seeds of *Brassica campestris* L. ssp. *pekinensis* [32], in a chloroform extract of leaves of *Piper betle* [33], and also in those cases recognized based on Wiley mass database. Other minor components were identified, again by comparison with the mass spectra present in the aforementioned library, such as β-sitosterol (in **SCO** and **SCH**), γ-sitosterol and ergost-5-en-3-ol (in **SCO**) but this issue requires deeper investigation.

We are aware that with the method we chose (GC–MS) to analyze the extracts, some non-volatile polar components might be not eluted. To check whether this is the case, some amount of each extract was dissolved in CDCl_3_ and the respective ^1^H NMR spectrum was recorded at 600 MHz. From the spectra (see Figures S8 and S9 in the Supplementary Material), the presence of signals in the aromatic region can be noted, mainly belonging to benzoic acid; and signals in the range 4.0–5.4 ppm are likely due to vinylic and allylic protons of unsaturated fatty acids, even if a negligible amount of signals due to polar compounds such as glycosides cannot be completely ruled out. The main signals fall between 0.6 and 2.9 ppm, confirming the presence of a number of aliphatic and α-methylenic groups.

It has to be emphasized that, unexpectedly, paeonol was not detected, also when we subjected the roots of the two cultivars to extraction in other solvents (see Supplementary Material), such as ethyl acetate and methanol; in all cases, the absence of paeonol both in GC–MS chromatograms and in ^1^H NMR spectra was ascertained by comparison with an authentic commercial sample. Paeonol has been widely studied for its pharmacological properties, including anticancer activity; it has been found in roots of many *Paeonia* species, such as *P. suffrutticosa*, *P. clusii*, *P. mascula ssp. hellenica*, *P. parnassica*, *P. lactiflora* [17]. However, a study on the genetic and chemical characterization of white and red peony roots derived from *Paeonia lactiflora* revealed that the presence (or not) and the content of paeonol might depend on many factors, such as subspecies, region, and growth conditions (wild or cultivated) [15].

### 2.2. Biological Activity

#### 2.2.1. Effects of **SCH** and **SCO** on Cell Proliferation

**Figure 2:**
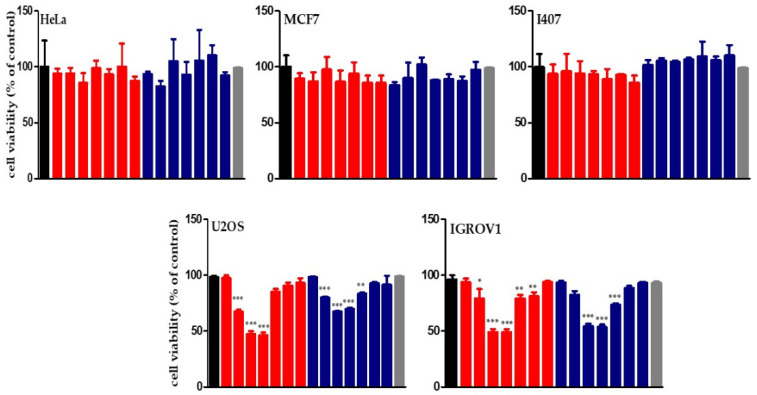
Effect of chloroformic extracts on the proliferation of neoplastic and normal human cell lines. HeLa, MCF7, I407, U2OS or IGROV1 cells were incubated for 24 h with increasing **SCH** (red) or **SCO** (blue) concentrations (1.25, 2.5, 5, 25, 50, 100, 250 μg/mL). The black bar corresponds to control cells; the gray bar to IGROV1 treated with DMSO 0.5%. Proliferation was evaluated by the MTT test, as reported in Materials and Methods. Results are expressed as means ± SD of three independent experiments. Statistical analysis was performed by Dunnett’s multiple comparison test following one-way ANOVA. * *p* < 0.05, ** *p* < 0.01 *** *p* < 0.001, significantly different from control cells.

**Figure 3:**
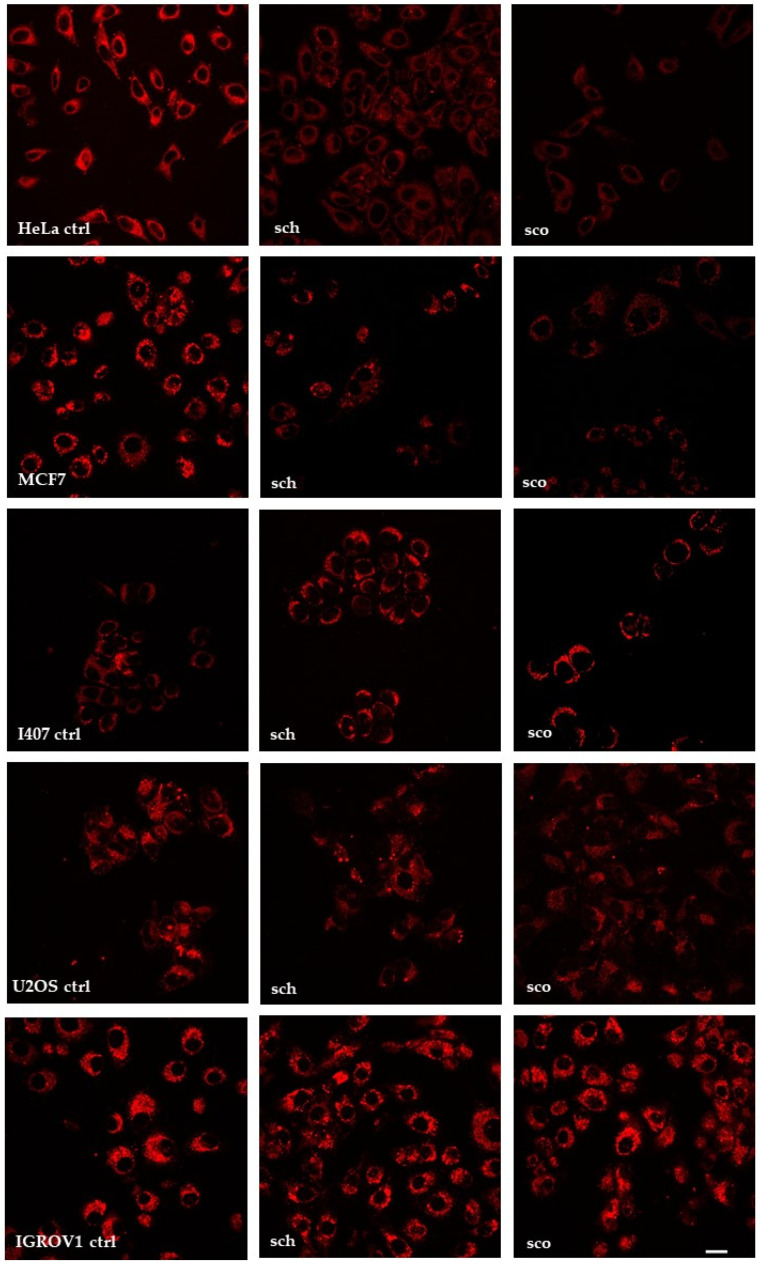
Confocal microscopy of cells after 1 h of treatment with **SCH** or **SCO**. Photographs were taken at 60× magnification, bar = 20 µm.

The in vitro cell growth inhibitory efficacy of **SCH** or **SCO** was determined by incubating HeLa, MCF7, I407, U2OS, and IGROV1 cells with increasing concentrations of the chloroformic extracts (from 1.25 μg/mL to 250 μg/mL) for 24 h. Cell proliferation is reported in Figure 2. **SCH** and **SCO** appeared to be well tolerated in HeLa, MCF7, and I407 cells, because no detectable decrease in proliferation was observed after 24 h. These results could be due to poor-to-null intracellular uptake. For this reason, all cells were treated with **SCH** or **SCO** for 1 h, and stained with LipidTOX™, a dye that has an extremely high affinity for neutral lipid droplets and can be detected by fluorescence confocal microscopy (Figure 3). Treatment with the two extracts decreased the lipid content in HeLa and MCF7 cells, while in I407 no significant variation was observed. On the contrary, in U2OS and, especially, in IGROV1, it caused an accumulation of lipids inside the cells. At the concentration of 2.5 μg/mL, **SCH** induced a decrease in proliferation in U2OS and IGROV1 of about 30%, while **SCO** induced a decrease of about 20%. At the concentration of 5 μg/mL and 25 μg/mL, both chloroformic extracts showed a much higher toxicity against U2OS and IGROV1, reducing proliferation by about 50%.

As shown in Figure 2, the chloroformic extracts at higher concentrations had a minor effect on the IGROV1 and U2OS cells proliferation. This effect could be due to an increased aggregation of the lipid components with consequent less diffusion of the active molecules inside cells. This hypothesis is supported by the 1-(4-trimethylammoniumphenyl)-6-phenyl-1,3,5-hexatriene (TMA-DPH) fluorescence assay, which showed a noticeable increase in the probe fluorescence intensity, due to its entrapping in structured lipid aggregates (Figure S1).

Subsequent experiments were carried out by using IGROV1 treated with SCH or SCO at a concentration of 5 μg/mL respectively, and with a mixture of the two extracts at a concentration 2.5 μg/mL each.

#### 2.2.2. Effects of Chloroformic Extracts on Phenotypic Behavioral Traits of IGROV1

**Figure 4:**
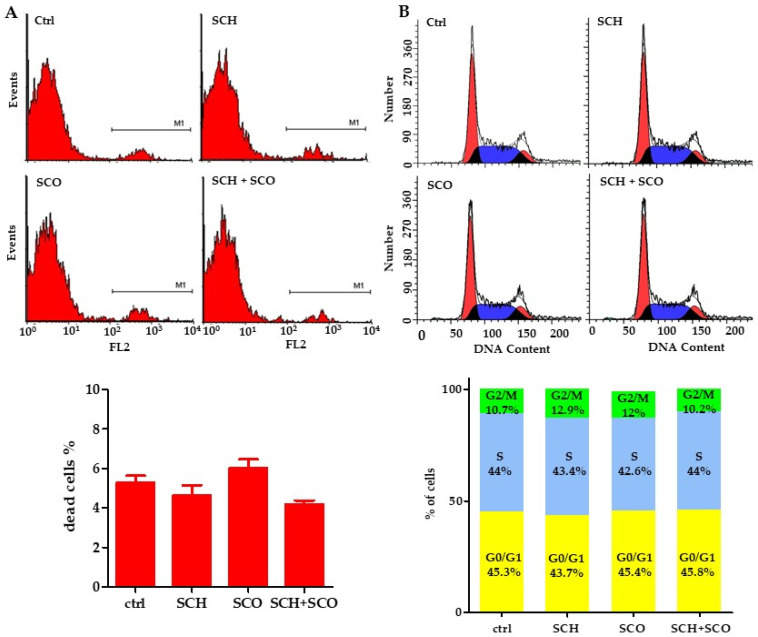
Effects of chloroformic extracts on cell viability and cell cycle phases in IGROV1. (**A**) Cell viability was tested by flow cytometry 24 h after treatment. Above: representative cytofluorimetric plots. Below: percentage of dead cells. Data are reported as mean ± SD of 3 independent experiments. (**B**) Cell cycle distribution. Above: cell cycle analysis by flow cytometry in control cells and in treated cells. Below: the column graph shows the percentage of cells in each cell cycle phase.

Cell viability is not affected by the treatments, as shown in Figure 4A, where the percentages of dead cells, evaluated by the cytofluorimetric assay of propidium iodide (PI), are reported. The effects on cell cycle are practically negligible, as evident in Figure 4B. This suggests that the lowering of cell proliferation is not due to cytotoxicity or to cell cycle perturbation. Therefore, our attention was switched to the effects on cellular phenotype.

In this preliminary study, quantitative phase imaging (QPI) was used to evaluate real time changes in cells proliferation, assessed as confluence and cell count.

**Figure 5:**
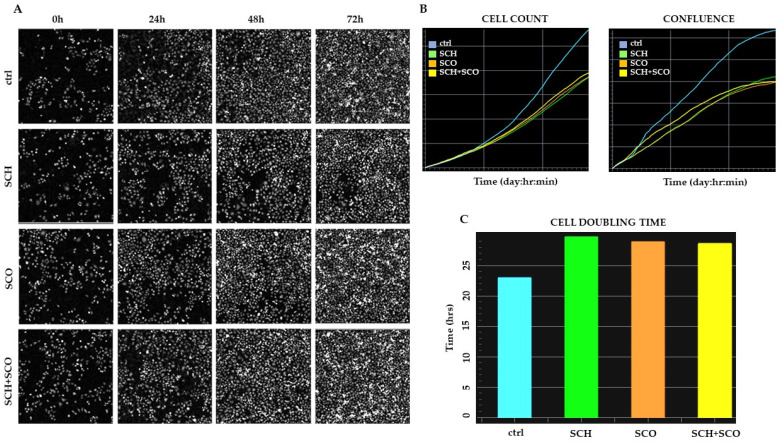
Quantitative phase imaging (QPI) and cell proliferation. (**A**) Representative image of QPI at 0, 24, 48, and 72 h of control and treated cells with 5 μg/mL **SCH** or **SCO**, or co-administration of **SCH** and **SCO** 2.5 μg/mL. (**B**) Plots of cell count and confluence over time for cells treated and untreated control. (**C**) Histogram plot illustrating median cell doubling time for cells treated and untreated control. Analysis of the growth curve was mediated on 6 wells for each treatment.

To this end, the Livecyte (Phasefocus) label-free time-lapse microscopy, based on ptychography, was used. This technique enables the evaluation of cellular morphology, cell count, dry mass, and confluence without labeling cells with dyes or antibodies. The cells are unperturbed, and the imaging process is not harmful, therefore not requiring the use of high energy lasers as in conventional microscopy [34,35]. In Figure 5A, the images of control and treated cells after 0, 24, 48, and 72 h of growth are reported. Cell count and confluence, and doubling time evaluated over 72 h of analysis are reported in Figure 5B,C, respectively.

As well as measuring changes in area-based cell confluence [36], QPI generates unique phase metrics, which provide accurate measurement of cell growth at an individual-cell level alongside growth and proliferation at a population level. Specifically, a cell’s dry mass can be calculated from the phase delay [37,38]. The increase in total dry mass of cells that occurs under normal growth and proliferation can be compared to the rate of change in total dry mass observed upon treatment (Figure 5A). Retardation or decrease in the rate of change is indicative of cytostatic or cytotoxic effects of a drug on a population [39]. It is evident that treatment with the *Paeonia* extracts induces a slowdown in growth, evaluated as cell counts and as confluence. No detached cells are evident even after 72 h, indicating that these phytoextracts are not cytotoxic, and confirming the data obtained by the cytofluorimetric assay of PI staining of dead cells. However, a clear increase in doubling time is observed, from 22 ± 0.6 to 30 ± 0.3 h for SCH treated cells and to 28 ± 0.3 h for SCO and SCH+SCO treated cells. In conclusion, these data indicate that the chloroformic extracts induce a cytostatic effect in IGROV1.

#### 2.2.3. Assessment of Mitochondrial Membrane Potential and Reactive Oxygen Species (ROS) Levels in IGROV1 Treated with Chloroformic Extracts

**Figure 6:**
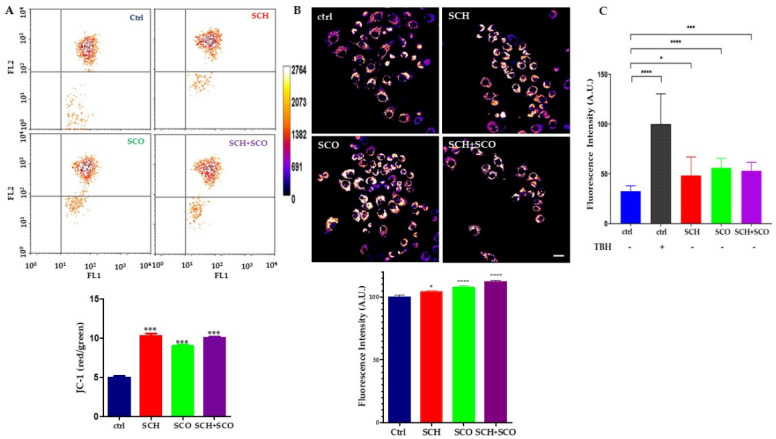
*Paeonia* extracts induce mitochondrial hyperpolarization and increase ROS level. (**A**) Above: IGROV1 cells treated with **SCH** or **SCO** or **SCH** + **SCO** for 24 h, stained with JC1 dye for 30 min at 37 °C, and analyzed using flow cytometer, reporting green fluorescence (FL1) of JC1 monomer in abscissa, and red fluorescence (FL2) of JC1 aggregates in ordinate. Results are represented as contour plots. A representative experiment of five is reported. Below: plot of ratio between red and green fluorescence. *** *p* < 0.001. (**B**) Above: representative fluorescence image of IGROV1 treated with *Paeonia* extracts for 24 h and then loaded with Tetramethylrhodamine methyl ester perchlorate (TMRM). On the left, the pseudo color intensity bar of TMRM fluorescence is shown, with white and black representing maximum and minimum intensity, respectively. Below: relative quantification of TMRM intensity. Results are given as means ± SD of three independent experiments and are compared to controls, taken as 100%. (*) *p* < 0.05, and (****) *p* < 0.0001. (**C**) ROS were detected following DCF fluorescence. ROS were induced by 24 h treatment with *Paeonia* extracts or by 30 min exposure to 150 µM TBH in control IGROV1. Data are the mean ± S.D. and are expressed as arbitrary fluorescence units (A.F.U.) normalized on control + TBH. Asterisks refer to the statistically significant increase in ROS production in treated samples. (*n* = 6, * *p* < 0.05; *** *p* < 0.001; **** *p* < 0.0001).

The chloroformic extracts administered to IGROV1 are enriched in fatty acids that accumulate in droplets, as clearly shown in Figure 6. This observation led us to hypothesize that the accumulated lipids could increase lipid catabolism with consequent modification of the basal redox state.

Indeed, it is known that an increase in either lipid [40] or glucose [41] concentration, and of their oxidative catabolism, results in higher electron flux in the electron transfer chain by raising the level of electron donors NADH and FADH_2_ in the mitochondrial matrix. In particular, by raising the FADH_2_:NADH ratio, fatty acid beta-oxidation further increases the level of ubiquinol which is the reduced form of electron acceptor ubiquinone [Speijer]. The resulting hyperpolarization of the mitochondrial inner membrane potential partially inhibits electron transport in complex III and accumulates electrons on ubisemiquinone to generate ROS [41,42].

For these reasons, we firstly investigated the mitochondrial membrane polarization state (ΔΨm).

We are aware that the correct assessment of “mitochondrial hyperpolarization” is complex and susceptible to artifacts due to the utilization of fluorescent probes [43,44]. Therefore, we performed this assay by means of two different probes, the cationic carbocyanine dye JC-1, and the rhodamine-derived Tetramethylrhodamine methyl ester perchlorate (TMRM) [43]: they clearly demonstrate mitochondrial hyperpolarization after *Paeonia* extract treatments, as shown in Figure 6.

The metachromatic fluorescent dye JC-1 is characterized by a shift of fluorescent emission from green, when the dye is a monomer, to red, when it accumulates in active mitochondria and polymerizes. The flow cytometric plots are shown in Figure 6A. The ratio between the red and green fluorescence is an index of ΔΨm. The ratio in control cells is around five, and doubles after 24 h of *Paeonia* treatment for all the extracts (Figure 6A). The mitochondrial hyperpolarization was also confirmed by confocal microscopy analysis using TMRM, as shown in Figure 6B.

We then investigated the production of ROS using DCF-DA. Figure 6C shows the endogenous production of ROS in control and treated IGROV1. The lipid extracts induce a significant increase in ROS, but interestingly, the production of radicals does not reach cytotoxic levels such as those produced by TBH, a chemical inducer.

The ROS increase could be due to two different causes: (i) the increase in internalization of unsaturated fatty acids could lead to their peroxidation, resulting in higher ROS production [45]; (ii) the intracellular accumulation of lipids could stimulate their catabolism with consequent increase in reduced coenzymes into the mitochondrial matrix, leading to higher ROS levels [40]. Moreover, it has been reported that at high ΔΨm the mitochondrial respiratory chain becomes a significant producer of ROS and their generation also depends exponentially on ΔΨm [46,47,48]. Hyperpolarization of mitochondria, maintained for a long time, could potentially be harmful to these organelles and consequently to the cell, probably due to this concomitant increase in intracellular ROS [43].

## 3. Materials and Methods

### 3.1. Plant Material, Solvents and Chemicals

Roots of *Paeonia officinalis* “Rubra Plena” and *Paeonia* “Pink Hawaiian Coral” varieties were supplied by Società Agricola La Riniera (Castel San Pietro Terme, Bologna, Italy) and stored in a freezer from the time of delivery until the carrying out of the experiments. The plant material was authenticated by Dr. Lucia Romani and samples of plant material were deposited in the Herbarium Universitatis Bononiensis (BOLO) under voucher specimen numbers BOLO0602017 and BOLO0602018 for “Pink Hawaiian Coral” and “Rubra Plena” cultivars, respectively.

*Paeonia officinalis* “Rubra Plena” is a very ancient cultivar of *Paeonia officinalis*, and in the USA, it is known as Memorial Day Peony, because it was usually the only peony that flowered on that date. *Paeonia* “Pink Hawaiian Coral” (*Paeonia lactiflora* “Charlie’s White” x *Paeonia* “Otto Froebel”), is a hybrid of herbaceous peony obtained by Roy G. Klehm in 1981 and registered among the cultivars of the American Peony Society with the number 1981-239:29 [49]. “Charlie’s White” is a cultivar of *Paeonia lactiflora*. “Otto Froebel” is a hybrid, probably a cross between *Paeonia officinalis* and *Paeonia aretina*, obtained by a Mr. Peter Barr of London whose archive was destroyed by fire. However, in some texts it is described as a cultivar of *Paeonia peregrina*. It is certain that the propensity to produce adventitious buds from even small portions of rhizome seems very characteristic of *Paeonia officinalis* but, at least as far as we know, this has not yet been clarified with cytological or other investigations.

As for the aspect of the roots (Figure 1), those belonging to the “Rubra Plena” variety are dark brown in color, thick, fleshy, and not very filamentous; the roots of the “Pink Hawaiian Coral” are characterized by a more threadlike appearance, less thick, and with a greater presence of tendentially filamentous parts.

The solvents used and the paeonol, used for comparison, were purchased from Sigma-Aldrich (Milano, Italy). The nuclear magnetic resonance spectra were performed with a Varian Inova 600 spectrometer (Varian, Palo Alto, CA, USA) and the chemical shift δ is given in ppm, taking the solvent as a reference (δ = 3.31 or 7.26 for ^1^H-NMR in CD_3_OD or CDCl_3_, respectively). Gas chromatography–mass spectrometry analysis was performed using a Hewlett-Packard HP 6890 gas chromatograph (Hewlett-Packard, Ramsey, MN, USA) directly interfaced with a mass selective detector Agilent 5973 Network (Agilent Technology, Santa Clara, CA, USA). Injector temperature: 250 °C (mode: split with 50:1 splitting ratio). Column: Agilent Technology VF-WAXms, length: 30 m; diameter: 0.25 mm; film thickness: 0.25 μm. Oven temperature was programmed as follows: 60 °C for 5 min, increased up to 260 °C at the rate of 10 °C·min^−1^, followed by isotherm at 260 °C for 30 min; solvent delay time: 4.0 min. The carrier gas was helium, with an initial flow rate of 1 mL/min; transfer line temperature was 280 °C; the ionization was obtained by electron impact (EI), acquisition range 50–500 *m*/*z*.

### 3.2. Extraction Procedure

Roots of *Paeonia* variety *officinalis* “Rubra Plena” (995 g) and “Pink Hawaiian Coral” (993 g) were defrosted, washed with water, then finely cut using a domestic vegetable grinder. The roots were placed in an oven at 70 °C in the dark until constant weight, then were pulverized using a spice mill.

Masses of 375 g and 340 g of dried root were obtained from “Rubra Plena” and “Pink Hawaiian Coral”, respectively.

A total of 300 g of each sample were distributed in the cellulose thimble of three Soxhlet extractors (100 g in each apparatus) equipped with a condenser. The three extractors were connected in parallel (see Figure S2 in Supplementary Material). Chloroform (500 mL) was inserted in a round-bottomed flask connected to each extractor. Heating mantles were then placed under the flasks, adjusted to a temperature slightly higher than the boiling point of the solvent, and extraction began. The roots were left under extraction for 24 h, the system was allowed to cool down to room temperature, then the solutions derived from the three extractors were collected and the solvent was removed in vacuo until a constant weight of the residue was obtained: 1.71 g of **SCO** and 1.85 g of **SCH**; in both cases the yield was 0.6% compared to the weight of the dried roots. A mass of 100 mg of each sample were dissolved in 2.5 mL of chloroform, and 0.5 μL from each were taken and analyzed by GC–MS.

### 3.3. Cell Culture and Treatments

The human cervical cancer (HeLa), human breast cancer (MCF7), normal human intestine (I407), human bone osteosarcoma (U2OS) cell lines were purchased from American Type Culture Collection (ATCC, Manassas, VA, USA); the human ovarian cancer cell line (IGROV1) was kindly provided by the Istituto Nazionale Tumori (IRCCS, Milano, Italy). Cells were cultured in RPMI 1640 medium (Labtek Eurobio, Milan, Italy), supplemented with 10% FCS (Euroclone, Milan, Italy) and 2 mM L-glutamine (Sigma-Aldrich, Milan, Italy), at 37 °C, and a 5% CO_2_ atmosphere. The dried compounds were dissolved in DMSO (Sigma-Aldrich, Milan, Italy) in a 10 mg/mL stock solution. In cell treatments, the final DMSO concentration never exceeded 0.5%.

#### 3.3.1. MTT Assay

Cells were seeded in 1.5 × 10^4^ cells/well in a 96-well culture plastic plate (Orange Scientific, Braine-l’Alleud, Belgium), and after 24 h of growth were exposed to increasing concentrations of **SCH** or **SCO** (from 1.25 μg/mL to 250 μg/mL) solubilized in RPMI 1640 medium. For measurement, the culture medium was replaced with 0.1 mL of 3-(4,5-dimethylthiazolyl-2)-2,5-diphenyltetrazolium bromide (MTT, Sigma-Aldrich, Milan, Italy) dissolved in PBS at the concentration of 0.2 mg/mL, and samples were incubated for 2 h at 37 °C. The blue–violet formazan salt crystals formed were dissolved in 0.1 mL of isopropyl alcohol for 20 min. The absorbance at 570 nm was measured using a multi-well plate reader (Tecan, Männedorf, CH), and data were analyzed by Prism GraphPad software, and expressed as % of controls (untreated cells).

#### 3.3.2. Fluorescence Measurements

Fluorescence spectra of 5 μg/mL or 250 μg/mL solution of **SCH** or **SCO** in DPBS (Sigma-Aldrich, Milan, Italy) were scanned. A few microliters of (TMA-DPH) (Thermo Fisher Scientific, Waltham, MA, USA) stock solution was added to the extracts solution in order to obtain a final probe concentration of 10 μM. The fluorescence intensity measurements were performed at room temperature, under moderate stirring, using a PTI QuantaMaster spectrofluorimeter (Photon Technology International, North Edison, NJ, USA) set at 360 nm for excitation and 440 nm for emission, with a 10 nm bandwidth. The background level was measured on unlabeled controls.

#### 3.3.3. Confocal Microscopy

Cells were grown on glass coverslips and exposed to **SCH** or **SCO** 5 µg/mL for 24 h. After treatment, cells were incubated with HCS LipidTOX™ Neutral Lipid (Thermo Fischer Scientific, Waltham, MA, USA), according to the manufacturer’s instructions. Specimens were embedded in Mowiol (Hoechst, Frankfurt, Germany), and multiple images were acquired by using sequential laser excitations at 568 nm. The images were collected by using a Nikon C1s confocal laser-scanning microscope (Nikon, Tokyo, Japan), equipped with a Nikon PlanApo 60X, 1.4-NA oil immersion lens.

#### 3.3.4. Cell Cycle Analysis and Quantitative Phase Imaging (QPI) Microscopy

For cell cycle analysis, IGROV1 cells were treated for 24 h with **SCH** or **SCO** 5 µg/mL or **SCH** 2.5 μg/mL + **SCO** 2.5 μg/mL for 24 h, detached with 0.11% trypsin (Sigma-Aldrich, St. Louis, MO, USA)/0.02% ethylenediaminetetraacetic acid (EDTA) (Sigma-Aldrich, St. Louis, MO, USA), washed in PBS (Sigma-Aldrich, St. Louis, MO, USA), and centrifuged. Cells were resuspended in citrate hypotonic buffer and stained with propidium iodide (PI) according to Micheletti et al. [50]. PI red fluorescence was acquired on a linear scale and analyzed by Modfit software (Verity, Topsham, ME, USA). Flow cytometric assays were performed on a Brite HS flow cytometer (BioRad, Hercules, CA, USA) equipped with a Xe/Hg lamp.

For QPI analysis, cells were seeded in a 96-well plate (Corning, Tewksbury, MA, USA) at 4 × 10^3^ per well. After 24 h, cells were treated with **SCH** or **SCO** 5 μg/mL or **SCH** 2.5 μg/mL + **SCO** 2.5 μg/mL in six replicates and then QPI imaging was performed using a Livecyte microscope (Phase Focus, Sheffield, UK). QPI images were acquired every 60 min for 3 days using a 10× objective (0.25 NA), at 37 °C and 5% CO_2_. QPI data were analyzed using Cell Analysis Toolbox software (Phase Focus, Sheffield, UK) to evaluate confluence and doubling times.

#### 3.3.5. Cell Viability

To assess cell viability, the PI exclusion assay was used. PI is not permeable to viable cells with intact membrane; only dead or dying cells appear stained. Briefly, detached IGROV1 cells were incubated in the dark for 1 min with PI 5 μg/mL in complete medium, and then analyzed by flow cytometry, acquiring the PI red fluorescence on a logarithmic scale.

#### 3.3.6. Measurement of Intracellular ROS Level

Reactive oxygen species were detected in intact cells, according to Bergamini et al. [51]. Briefly, IGROV1 were seeded at the density of 1.5 × 10^4^ cells per well in a 96-well plate and incubated for 24 h to allow adhesion. Then, cells were treated with **SCH** or **SCO** 5μg/mL or **SCH** 2.5 μg/mL + **SCO** 2.5 μg/mL for 24 h. Cells were then incubated with 10 µM DCFDA (Thermo Fisher Scientific, Waltham, MA, USA) for 1 h. To induce oxidative stress, cells were exposed to 150 µM tert-butyl hydroperoxide (TBH) in PBS for 30 min. Cells were then washed twice with PBS and the fluorescence emission from each well was measured (λexc = 485 nm; λem = 535 nm) with a multi-plate reader (Enspire, Perkin Elmer, Monza, Italy). Data are reported as the mean ± SD of at least three independent experiments.

#### 3.3.7. Mitochondrial Membrane Potential Measurement

Mitochondrial membrane potential was evaluated by using two experimental methods: (1) Cells were grown in 25 cm^2^ plates (Sarstedt AG, Sevelen, CH) at a density of 20 × 10^3^ cells/cm^2^ in complete culture media. Cells were treated with **SCH** or **SCO** 5 μg/mL or **SCH** 2.5 μg/mL + **SCO** 2.5 μg/mL for 24 h, and then with 2 µM JC-1 (Thermo Fisher Scientific, Waltham, MA, USA) dissolved in complete medium for 15 min at 37˚C. Then, cells were washed with PBS and the fluorescence emission was detected with a flow cytometer Brite HS: excitation wavelength was set at 475 nm for simultaneous aggregate and monomer excitation; emission wavelength was set at 530 nm (for monomer species determination) and 590 nm (for aggregate species determination). The results are expressed calculating the ratio between red and green emissions. (2) Alternatively, the mitochondrial transmembrane potential (ΔΨm) was assessed using the cationic dye TMRM (Thermo Fisher Scientific, Waltham, MA, USA). Cells were seeded in 8-well µ-slides (Ibidi, GmbH, Planegg/Martinsried, Germany) following the manufacturer’s instructions. Cells were treated with **SCH** or **SCO** 5 μg/mL or **SCH** 2.5 μg/mL + **SCO** 2.5 μg/mL for 24 h. Subsequently, cells were incubated with 50 nM TMRM dissolved in DMEM for 30 min. Then, cells were washed with HBSS (Sigma-Aldrich, St. Louis, MO, USA) and analyzed by confocal microscopy. Images were acquired on a Nikon C1 confocal microscope (see above) and the fluorescence intensity analysis was performed using the ImageJ software standard tool. A manual threshold was applied to the red channel in order to remove the background noise. At least two randomly chosen fields were acquired for each condition and the mean signal intensity was measured for single cell.

#### 3.3.8. Statistical Analysis

MTT, DCFDA, TMRM, JC1, and QPI experiments were repeated at least three times, on three independent samples. One-way analysis of variance (ANOVA) followed by Dunnett’s multiple comparison test was used for repeated measurement values. Differences of *p* < 0.05 were considered significant. Statistical analysis was carried out using Prism GraphPad software.

## 4. Conclusions

To the best of our knowledge, the present study represents the first investigation on the biological effect of low-polar fraction of *Paeonia* roots extracts.

Roots of two cultivars of genus *Paeonia*, namely “Pink Hawaiian Coral” and *officinalis* “Rubra Plena”, have been extracted with chloroform and the biological effects of the obtained lipidic fraction were investigated. The two extracts showed similar activities when administered at concentrations up to 5 μg/mL, and when co-administered at 2.5 μg/mL. They revealed significant selectivity, being able to elicit harmful effects only in two cancer cell lines, the osteosarcoma U2OS and, to a higher extent, the ovarian cancer IGROV1. The most important biological effect reported in this study is a cytostatic effect which correlates with an increase in mitochondrial polarization and ROS production.

It is known that ROS increase could induce a signaling cascade that also leads to cellular stress in tumor cells. However, it has been demonstrated that dietary phytochemicals extracts have the capacity to increase ROS production in cancer cells, inhibiting cancer cell proliferation [52,53].

This study represents the starting point for an in-depth chemical study on the active components of *Paeonia* roots and on their biological effects at a more detailed level, as well as on the importance of the synergism between the different plant components in phytotherapy.

## Figures and Tables

**Table 1:** Relative % areas of the GC–MS peaks for common components identified in “Rubra Plena” (**SCO**) and “Pink Hawaiian Coral” (**SCH**) extracts.

Compound	r.t. (min.)	SCO Area (%)	SCH Area (%)
Phenol	17.8	0.05	0.2
Benzoic acid	21.8	18.4	28.2
Palmitic acid	25.4	15.3	16.8
Heptadecanoic acid	26.3	0.5	0.6
Palmitoleic acid	26.6	0.6	0.8
Stearic acid	27.3	0.9	1.2
Oleic acid	27.7	5.9	8.3
Linoleic acid	28.3	39.6	32.6
Methyl linolenate	29.3	5.8	3.8
Aplysteryl acetate ^a^	34.8	9.7	5.9
Vitamin E	52.4	3.4	1.6

^a^: In the case of **SCH**, the same similarity (87%) was also detected with 25-epiaplysterylacetate-1.

## Data Availability

Not applicable.

## Supplementary Materials

The following are available online. Figure S1: Fluoresce spectra of TMA-DPH; Figures S2–S7: Soxhlet extractors and GC–MS chromatograms of **SCO** and **SCH** and of samples extracted with ethyl acetate; Figures S8 and S9: copies of ^1^H NMR spectra of **SCH** and **SCO**.

## Sample Availability

The samples of compounds are not available from authors.
