# Supplementary material for: Root Extracts of Two Cultivars of Paeonia Species: Lipid Composition and Biological Effects on Different Cell Lines: Preliminary Results
Source: Molecules. 2021 Jan 27;26(3):655. doi: 10.3390/molecules26030655 (PMC7865396; doi:10.3390/molecules26030655)
Supplement: Supplementary file 1 [file molecules-26-00655-s001.pdf]

Root extracts of two cultivars of *Paeonia* species: lipid composition and biological effects on different cell lines. Preliminary results.

Natalia Calonghi[1]*, Giovanna Farruggia[1], Carla Boga[2]*, Gabriele Micheletti[2], Elena Fini,[2] Lucia Romani,[3] Dario Telese[2], Erika Faraci[1], Christian Bergamini,[1] Stefano Cerini[2], and Nicola Rizzardi[1]

[1] Department of Pharmacy and Biotechnology, University of Bologna, Bologna (Italy); (NC), giovanna.farruggia@unibo.it (GF), erika.faraci@hotmail.it (EF); christian.bergamini2@unibo.it (CB2), nicola.rizzardi2@unibo.it (NR)

2 Department of Industrial Chemistry 'Toso Montanari', Alma Mater Studiorum Università di Bologna Viale Del Risorgimento, 4 402136 Bologna Italy; (CB1), elena.fini2@studio.unibo.it (EF), dario.telese2@unibo.it (DT), stefano.cerini3@unibo.it (SC), gabriele.micheletti3@unibo.it (GM)

3. Via Riniera, 2043 40024 Castel San Pietro Terme Italy, luciaromani@virgilio.it (LR)

Supplementary Material

Content Page

Figure S1. Fluorescence spectra of TMA-DPH **S2**

Figure S2. Soxhlets assembled in parallel to extract *Paeonia* roots S2

Figures S3-S7. GC-MS chromatograms of **SCO** and **SCH** and of samples extracted with ethyl acetate. S**3-S7**

Figures S8-S9. Copies of ^{1}H NMR spectra of **SCO** and **SCH**. S**8-S9**

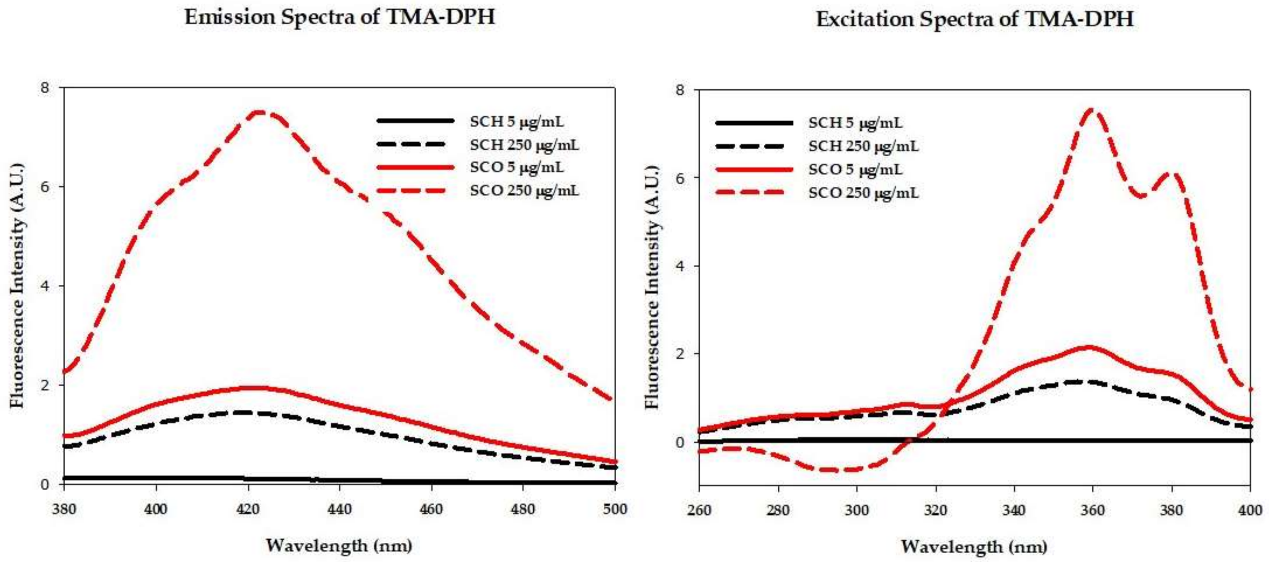

Figure S1. TMA-DPH fluorescence spectra in **SCH** (Black) and **SCO** (Red) at a concentration of 5µg/mL (continuous line) and at a concentration of 250µg/mL (dashed line). (left) Emission spectra of TMA-DPH recorded with the excitation set at 360 nm; (right) Excitation spectra of TMA-DPH recorded with the emission wavelength set at 430nm (leftt).

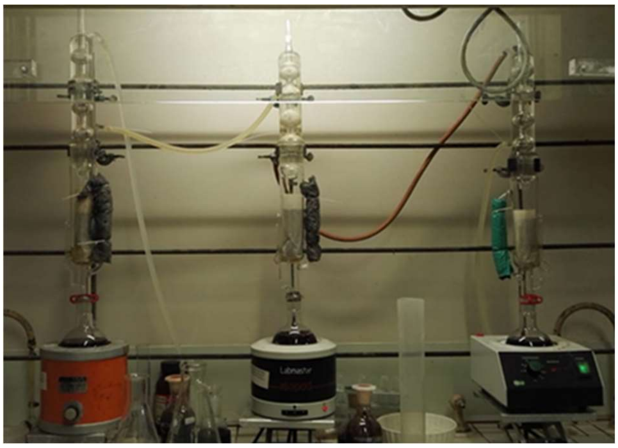

Figure S2. Soxhlets assembled in parallel to extract **SCH** with chloroform and, in a second time, also **SCO**.

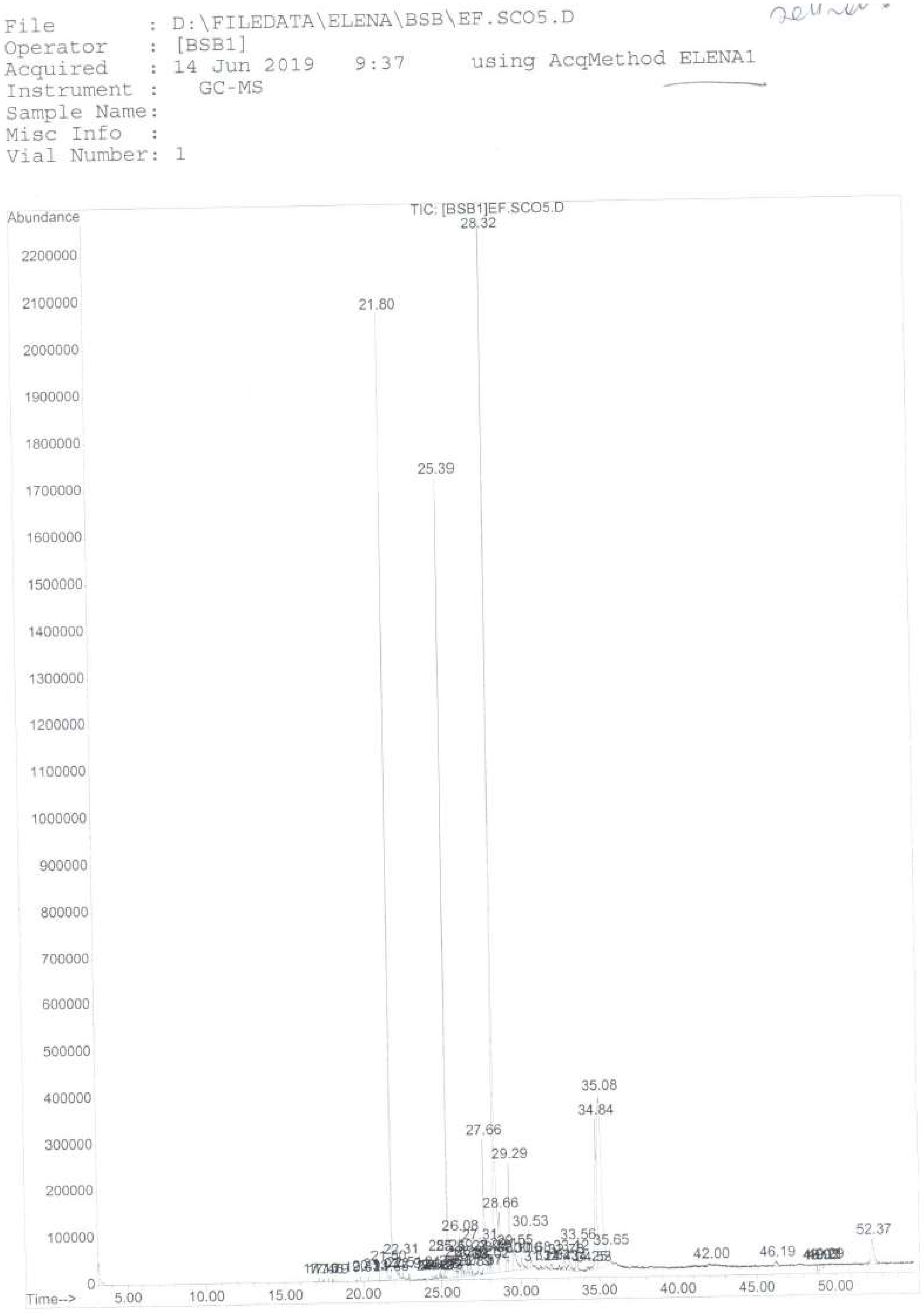

Figure S3. GC-MS chromatogram of **SCO**. Main peaks: r.t. = 21.80 min.: benzoic acid; r.t. = 25.39 min.: hexadecanoic acid; r.t. = 27.66 min.: oleic acid; r.t. = 28.32 min.: linoleic acid; r.t. = 29.29 min.: methyl linolenate; r.t. = 34.84 min.: aplysteryl acetate; r.t. = 35.08 min.: γ-sitosterol; r.t. = 52.37 min.: vitamin E.

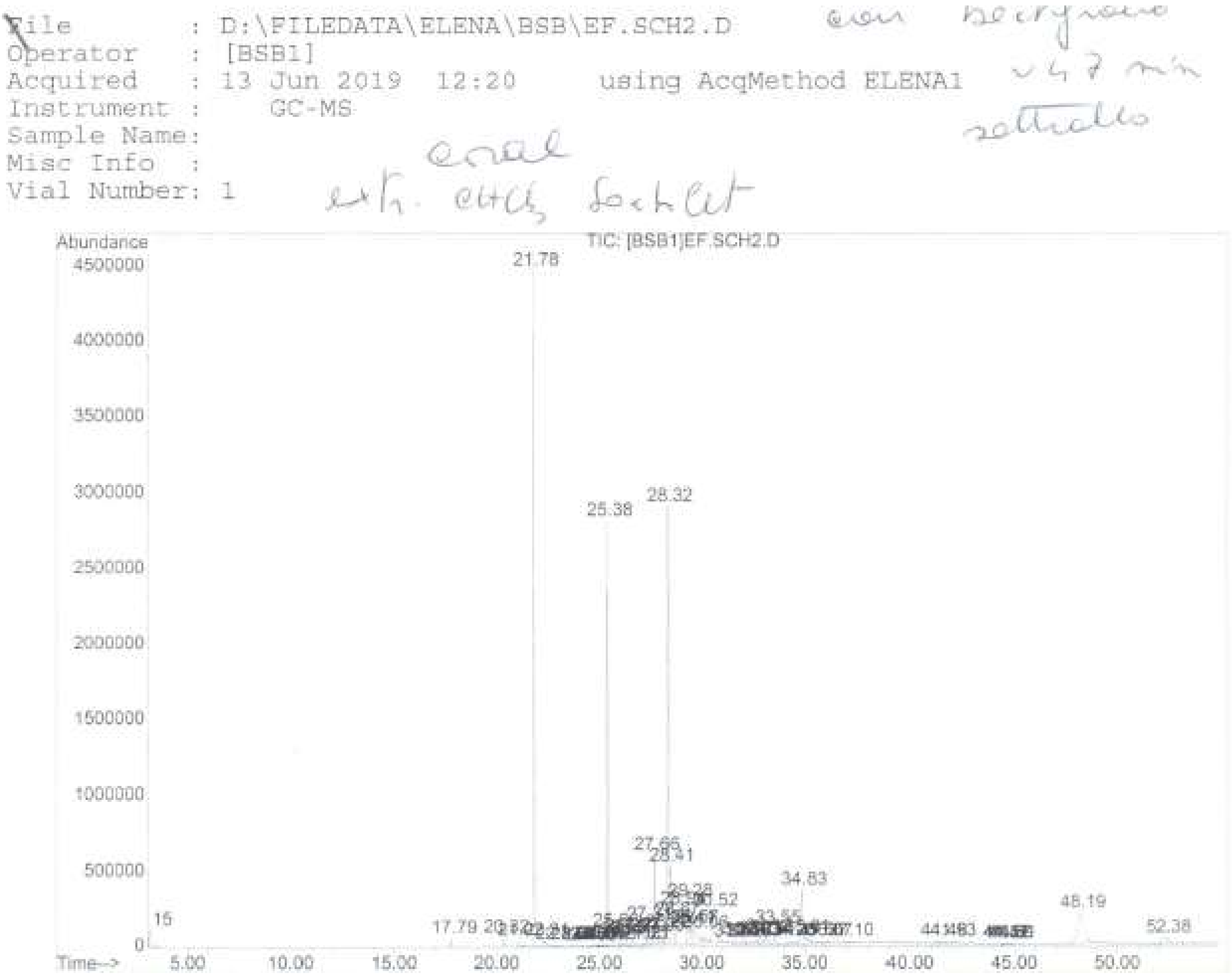

Figure S4. GC-MS chromatogram of **SCH.** Main peaks: r.t. = 17.79 min.: phenol; r.t. = 21.78 min.: benzoic acid; r.t. = 25.38 min.: hexadecanoic acid; r.t. = 27.66 min.: oleic acid; r.t. = 28.32 min.: linoleic acid; r.t. = 29.28 min.: methyl linolenate; r.t. = 34.83 min.: aplysteryl acetate; r.t. = 48.19 min.: β-sitosterol; r.t. = 52.38 min.: vitamin E.

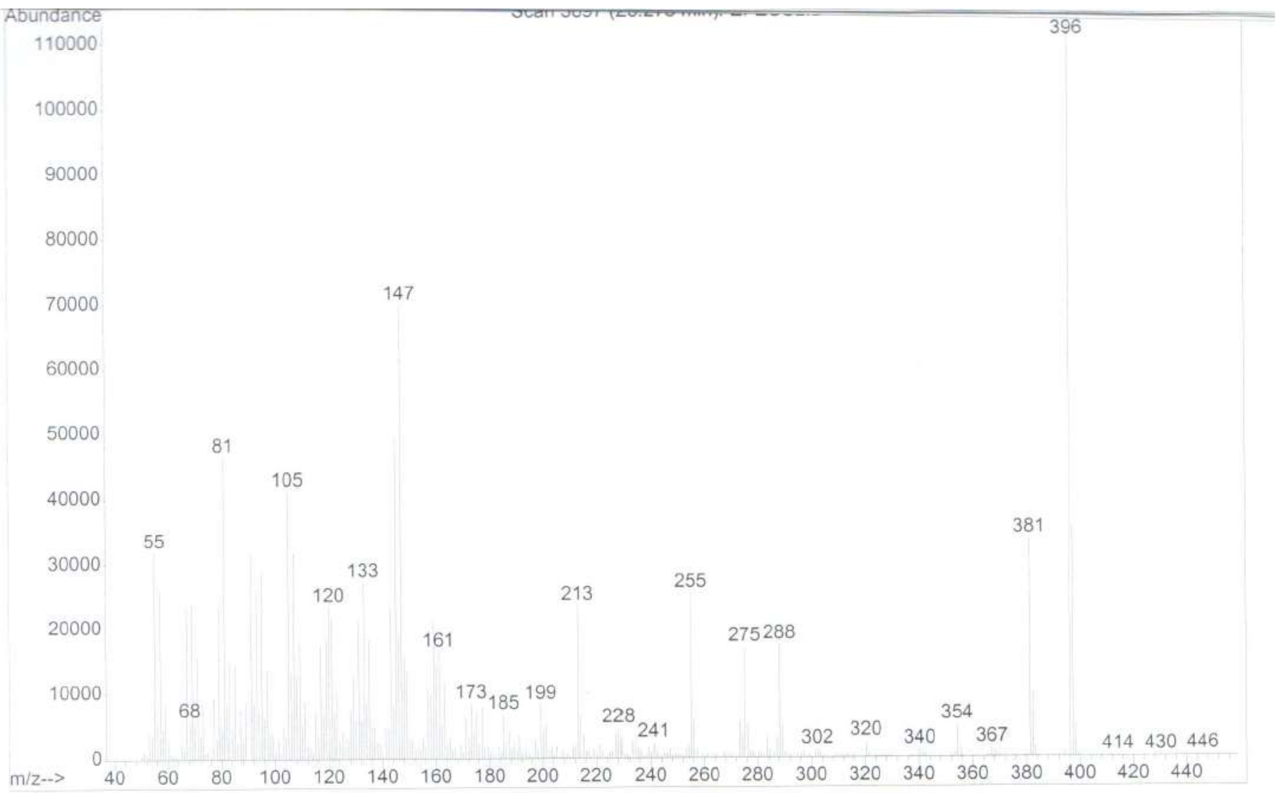

Figure S5. Mass spectrum recognized as belonging to aplysteryl acetate.

Extractions with ethyl acetate:

1. Powdered roots of *Paeonia Officinalis Rubra Plena* (75 g) were put in an Erlenmeyer flask and extracted with 200 mL of ethyl acetate at room temperature under magnetic stirring for 24 h. After decantation, the residue was subjected to a further extraction with 200 mL of ethyl acetate at room temperature under magnetic stirring for 24 h. After filtration, the combined organic layers were extracted with water (3 x 50 ml) and the organic layer was dried over anhydrous magnesium sulfate. After filtration and removal of the solvent *under vacuum* 0.57 g of residue were obtained (0.8%). 0.050 g of this extract are dissolved in 1 mL of dichloromethane and 0.5 µL were analyzed by GC-MS.
2. Powdered roots of *Paeonia Pink Hawaian Coral* (115 g) were inserted in an Erlenmeyer flask and extracted with 400 mL of ethyl acetate at room temperature under magnetic stirring for 24 h. After decantation, the residue was subjected to a further extraction with 400 mL of ethyl acetate at room temperature under magnetic stirring for 24 h. After filtration, the combined organic layers were extracted with water (3 x 100 ml) and the organic layer was dried over anhydrous magnesium sulfate. After filtration and removal of the solvent *under vacuum* 0.52 g of residue were obtained (0.5%). 0.050 g of this extract have been dissolved in 1 mL of dichloromethane and 0.5 µL were analysed by GC-MS.

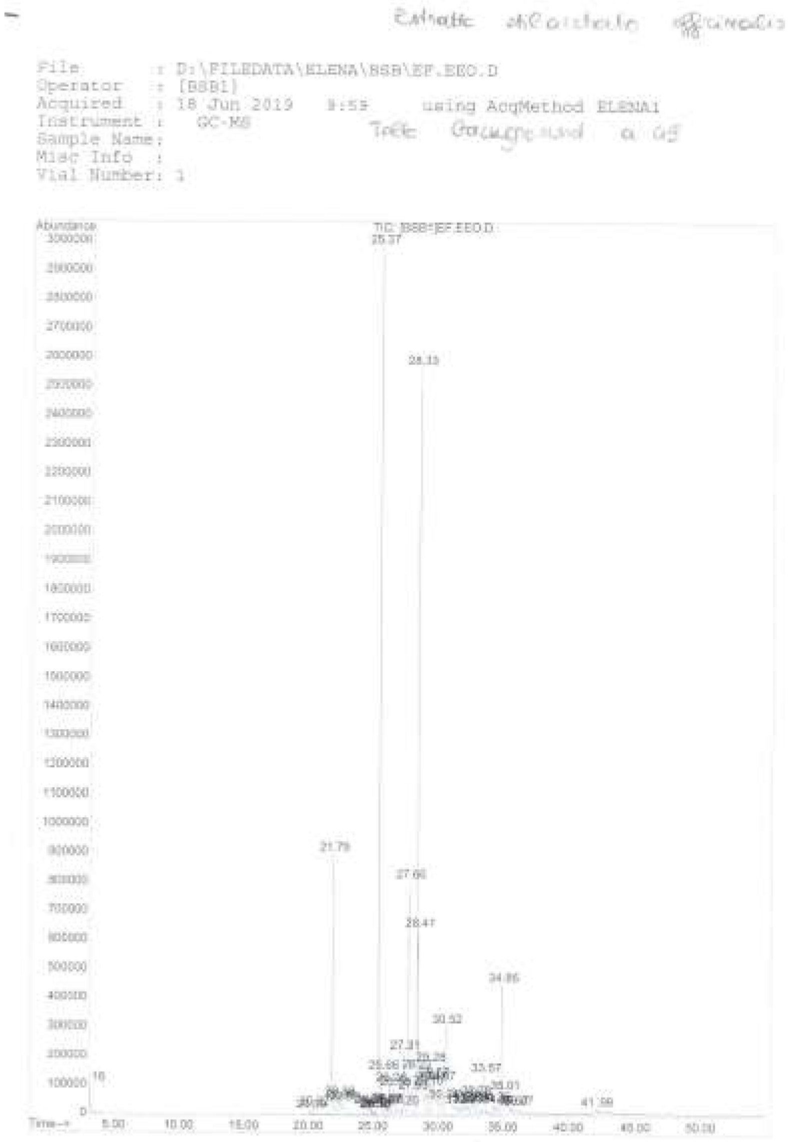

Figure S6. GC-MS chromatogram of ethyl acetate extract from roots of *Paeonia officinalis* ‘Rubra Plena’.

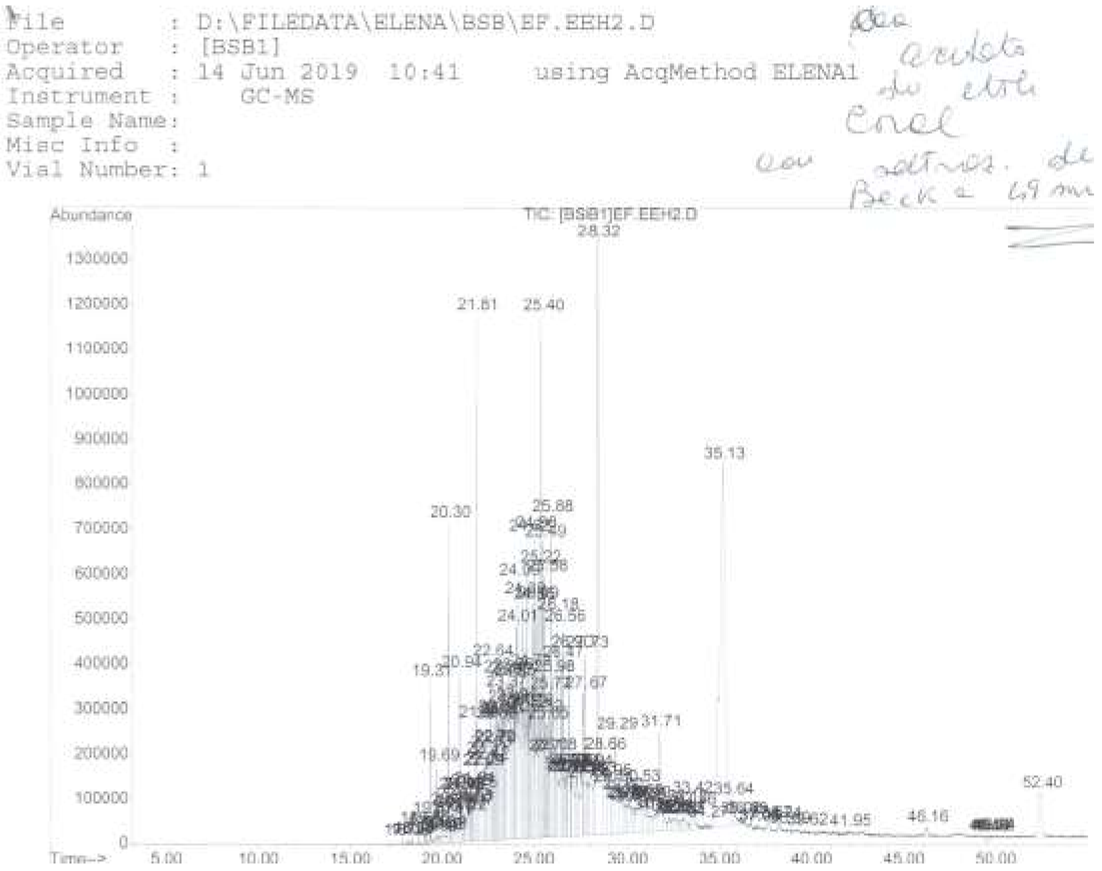

Figure S7. GC-MS chromatogram of the ethyl acetate extract from roots of *Paeonia* ‘Pink Hawaian Coral’.

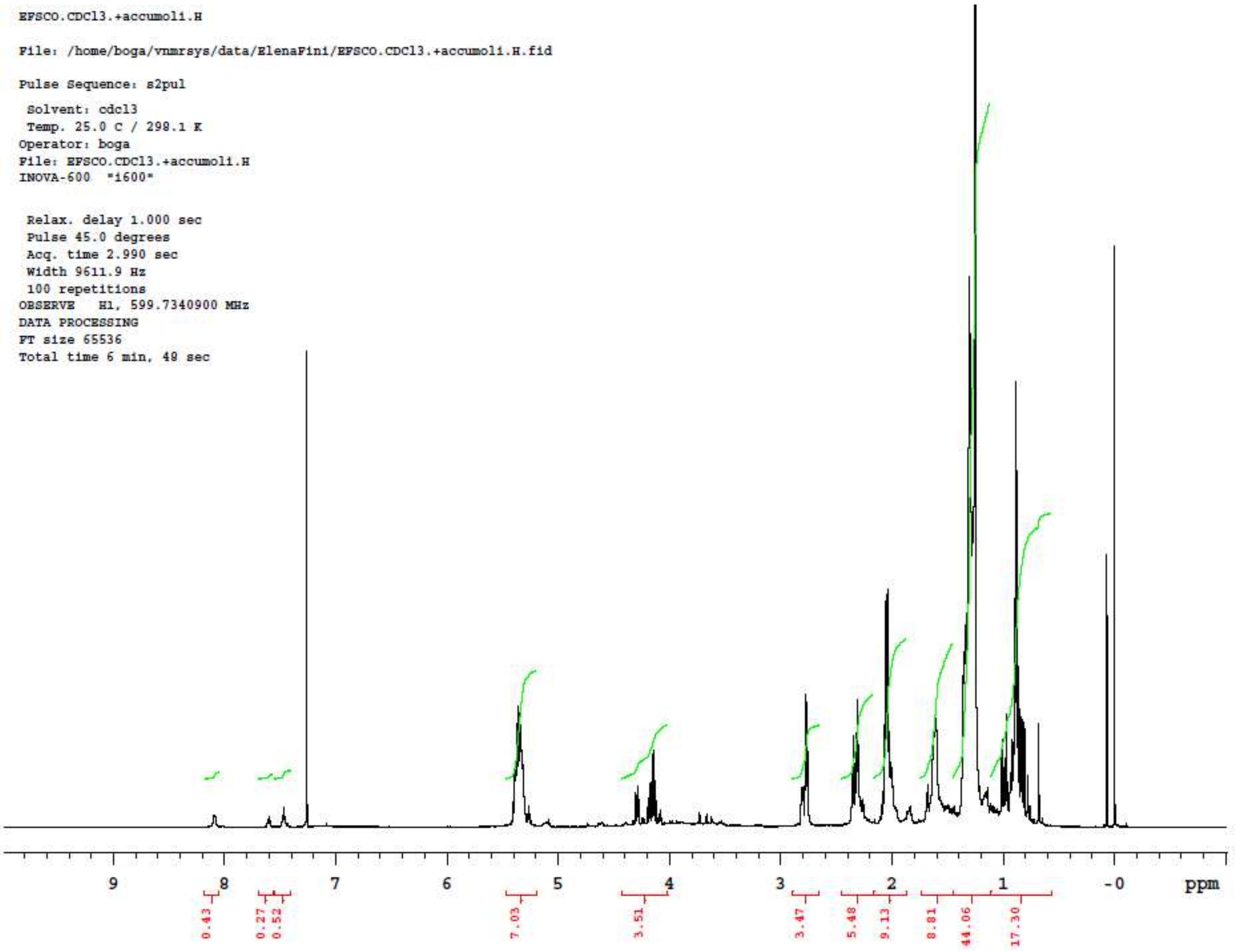

Figure S8. ^1H NMR spectrum (600 MHz, $CDCl_3$, 25 °C) of the extract **SCO** from roots of *Paeonia officinalis* ‘Rubra Plena’.

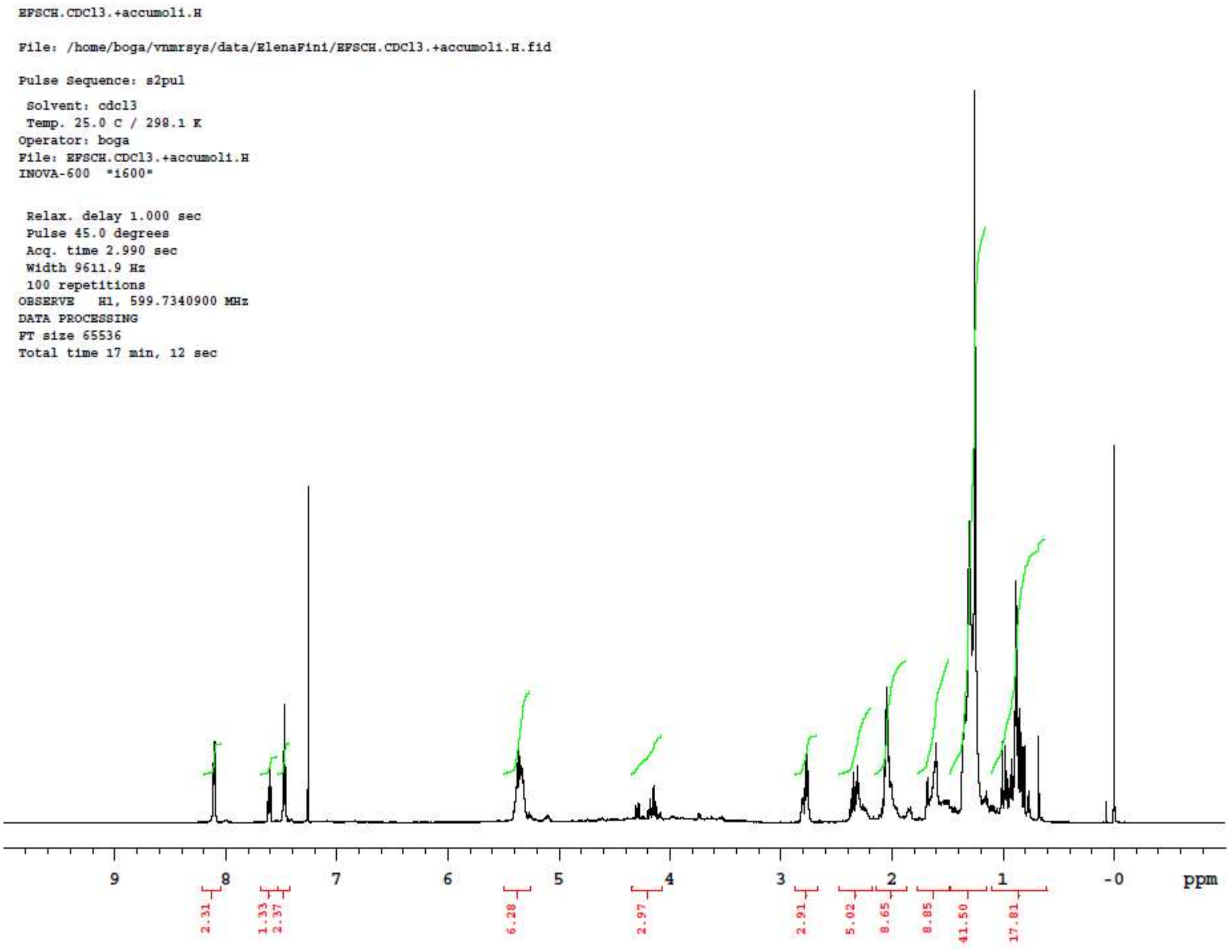

Figure S9. ^{1}H NMR spectrum (600 MHz, $CDCl_3$, 25 °C) of the extract **SCH** from roots of *Paeonia* ‘Pink Hawaian Coral’.
